# Supplementary material for: Copper(I)-Catalyzed Cross-Coupling of 1-Bromoalkynes with N-Heterocyclic Organozinc Reagents
Source: Molecules. 2022 Jul 17;27(14):4561. doi: 10.3390/molecules27144561 (PMC9315687; doi:10.3390/molecules27144561)
Supplement: Supplementary file 1 [file molecules-27-04561-s001.zip › molecules-1775357-supplementary.pdf]

Supplementary Materials

Copper(I) Catalyzed Cross-Coupling of 1-Bromoalkynes with N-Heterocyclic Organozinc Reagents

Christian Frabitore, Jérome Lépeule, and Tom Livinghouse

Department of Chemistry and Biochemistry, Montana State university, Bozeman, MT, 59717, USA

S1- ^{1}H NMR and ^{13}C NMR spectra of products **1a**, **2a**, **3a**, **1b**, and **2b**.

S2- ^{1}H NMR and ^{13}C NMR spectra of 1-bromo-1-octyne and 1-bromo-1-phenylethyne.

S1:

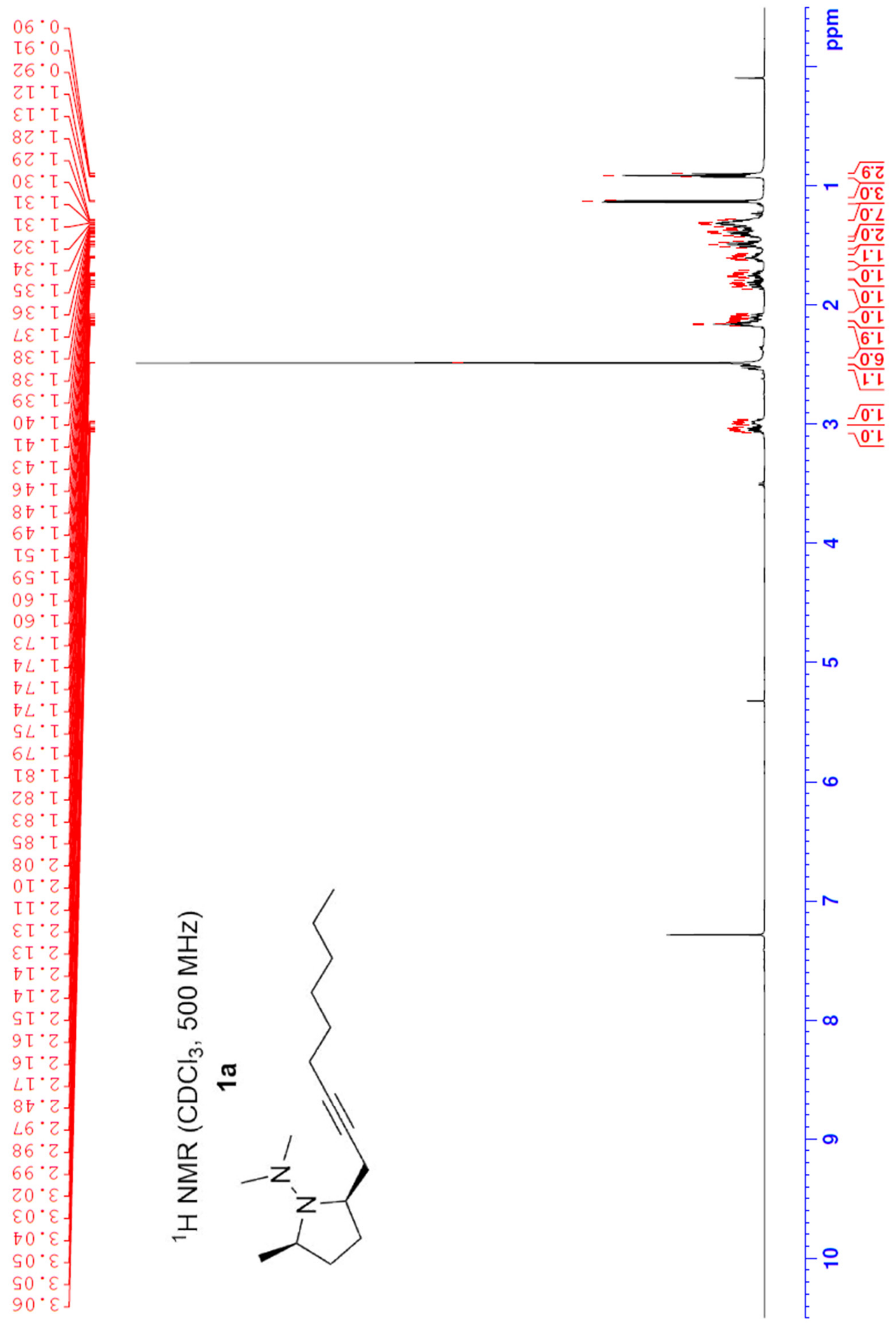

1H NMR (CDCl3, 500 MHz)
1a
N
N
ppm
0.90 0.91 0.92 1.12 1.13 1.28 1.29 1.30 1.31 1.31 1.32 1.34 1.35 1.36 1.37 1.38 1.38 1.39 1.40 1.41 1.43 1.46 1.48 1.49 1.51 1.59 1.60 1.60 1.73 1.74 1.74 1.74 1.75 1.79 1.81 1.82 1.83 1.85 2.08 2.10 2.11 2.13 2.13 2.14 2.14 2.15 2.16 2.16 2.17 2.48 2.97 2.98 2.99 3.02 3.03 3.04 3.05 3.05 3.06
2.9 3.0 7.0 2.0 1.1 1.0 1.0 1.0 1.0 1.9 6.0 1.1 1.0 1.0
1 2 3 4 5 6 7 8 9 10

^{13}C NMR ($CDCl_3$, 500 MHz)

1a

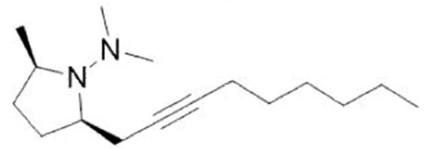

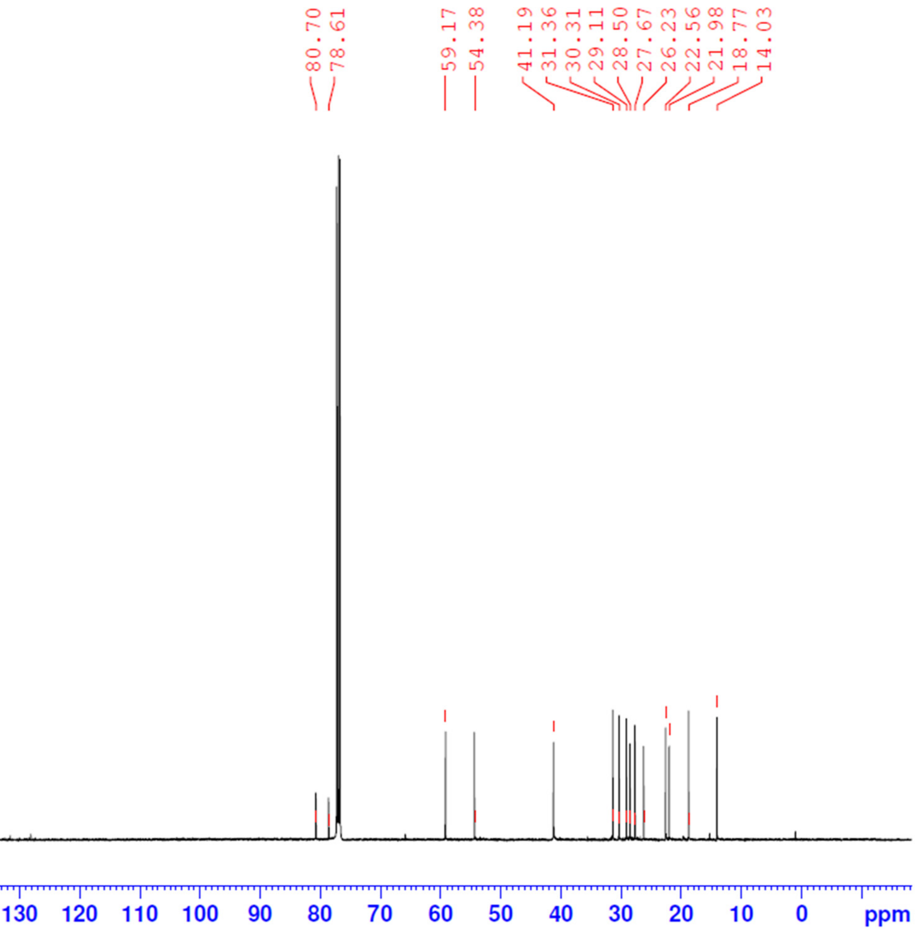

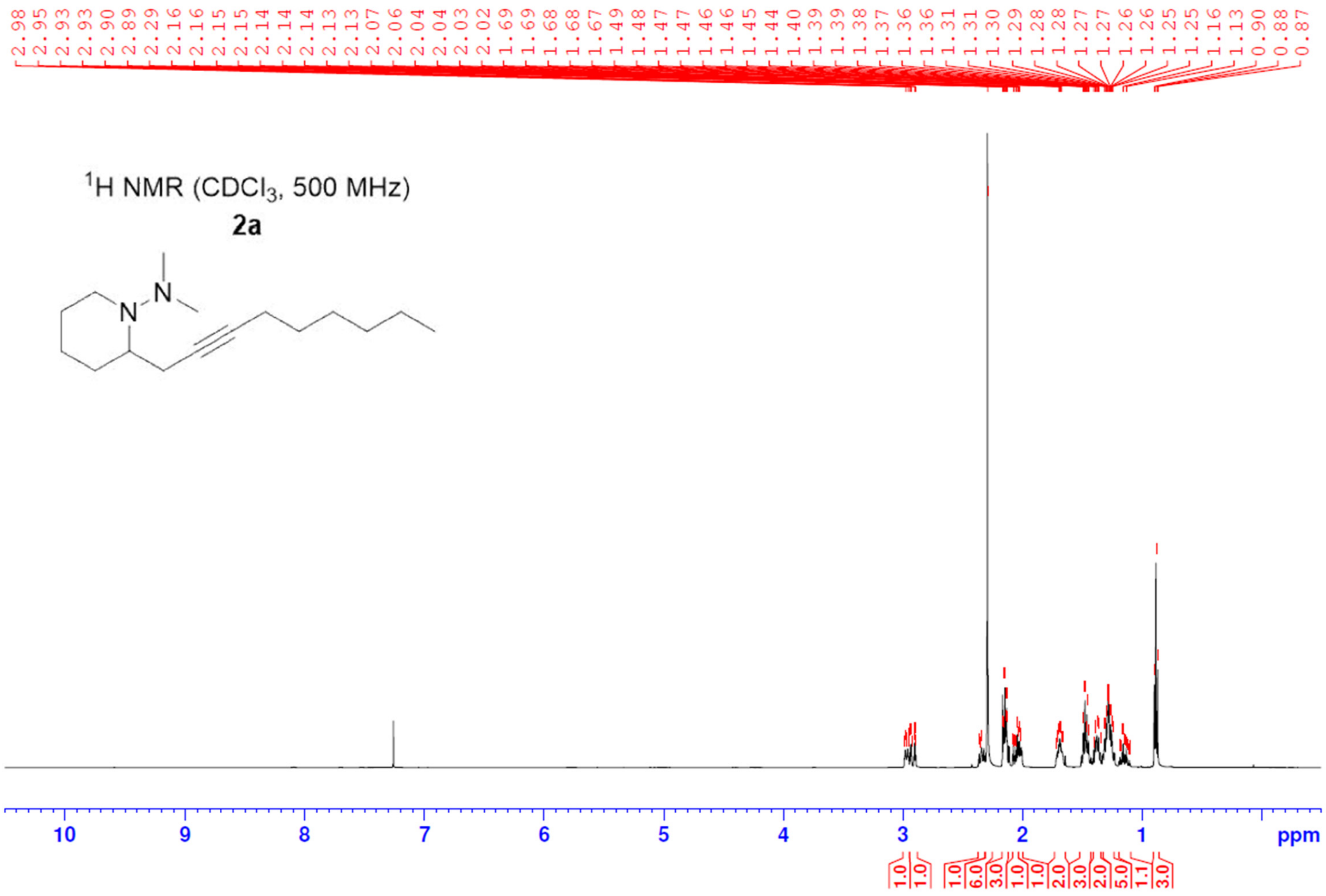

2.98
2.95
2.93
2.93
2.90
2.89
2.29
2.16
2.16
2.15
2.15
2.14
2.14
2.14
2.13
2.13
2.07
2.06
2.04
2.04
2.03
2.02
1.69
1.69
1.68
1.68
1.67
1.49
1.48
1.47
1.47
1.46
1.46
1.45
1.44
1.40
1.39
1.39
1.38
1.37
1.36
1.36
1.31
1.31
1.30
1.29
1.28
1.28
1.27
1.27
1.26
1.26
1.25
1.25
1.16
1.13
0.90
0.88
0.87
^{1}H NMR ($CDCl_3$, 500 MHz)
2a
N
N
10
9
8
7
6
5
4
3
2
1
ppm
1.0
1.0
1.0
6.0
3.0
1.0
1.0
2.0
3.0
2.0
5.0
1.1
3.0

^{13}C NMR ($CDCl_3$, 500 MHz)

2a

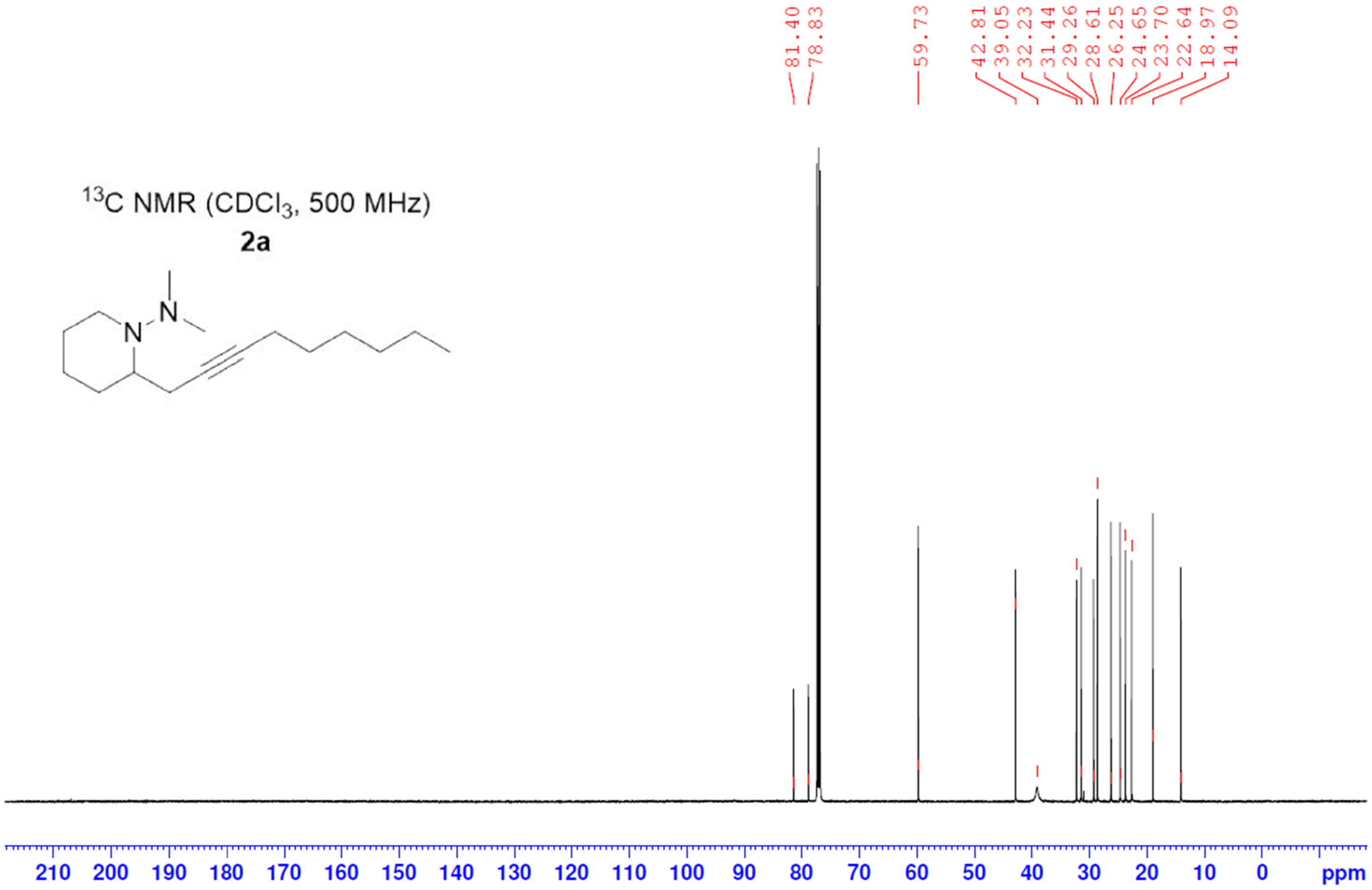

^{1}H NMR ($CDCl_3$, 500 MHz)

3a

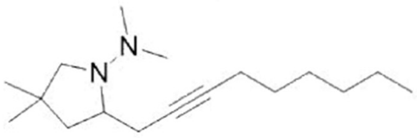

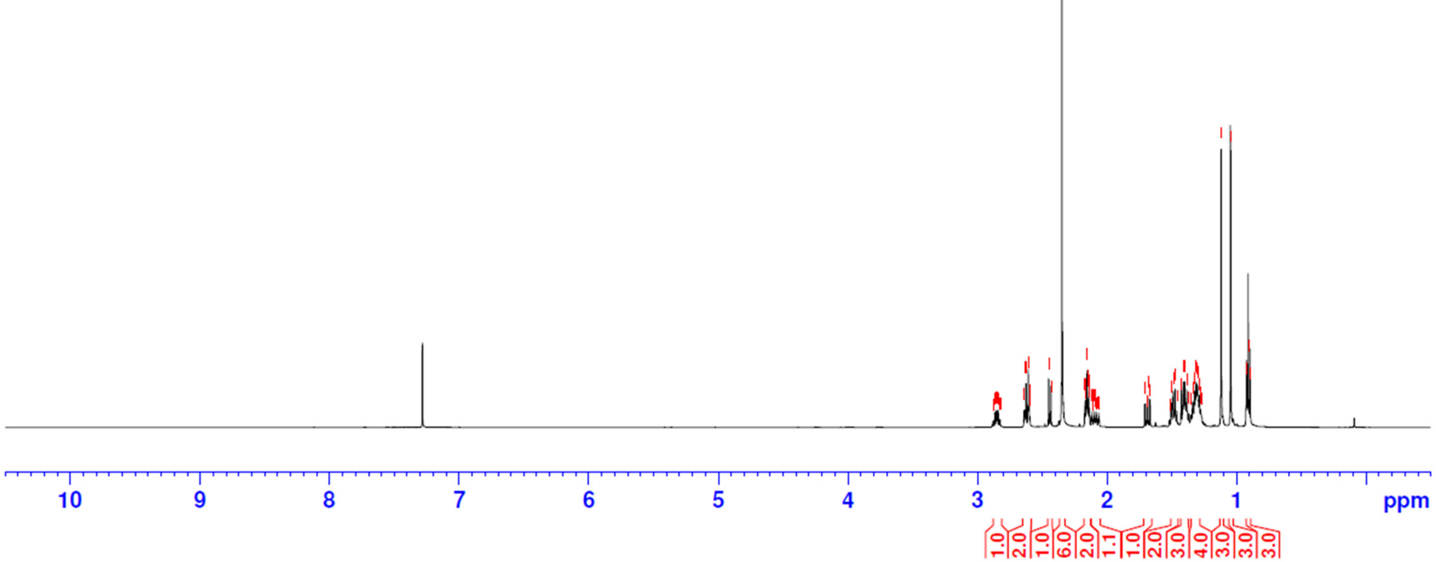

^{13}C NMR ($CDCl_3$, 500 MHz)

3a

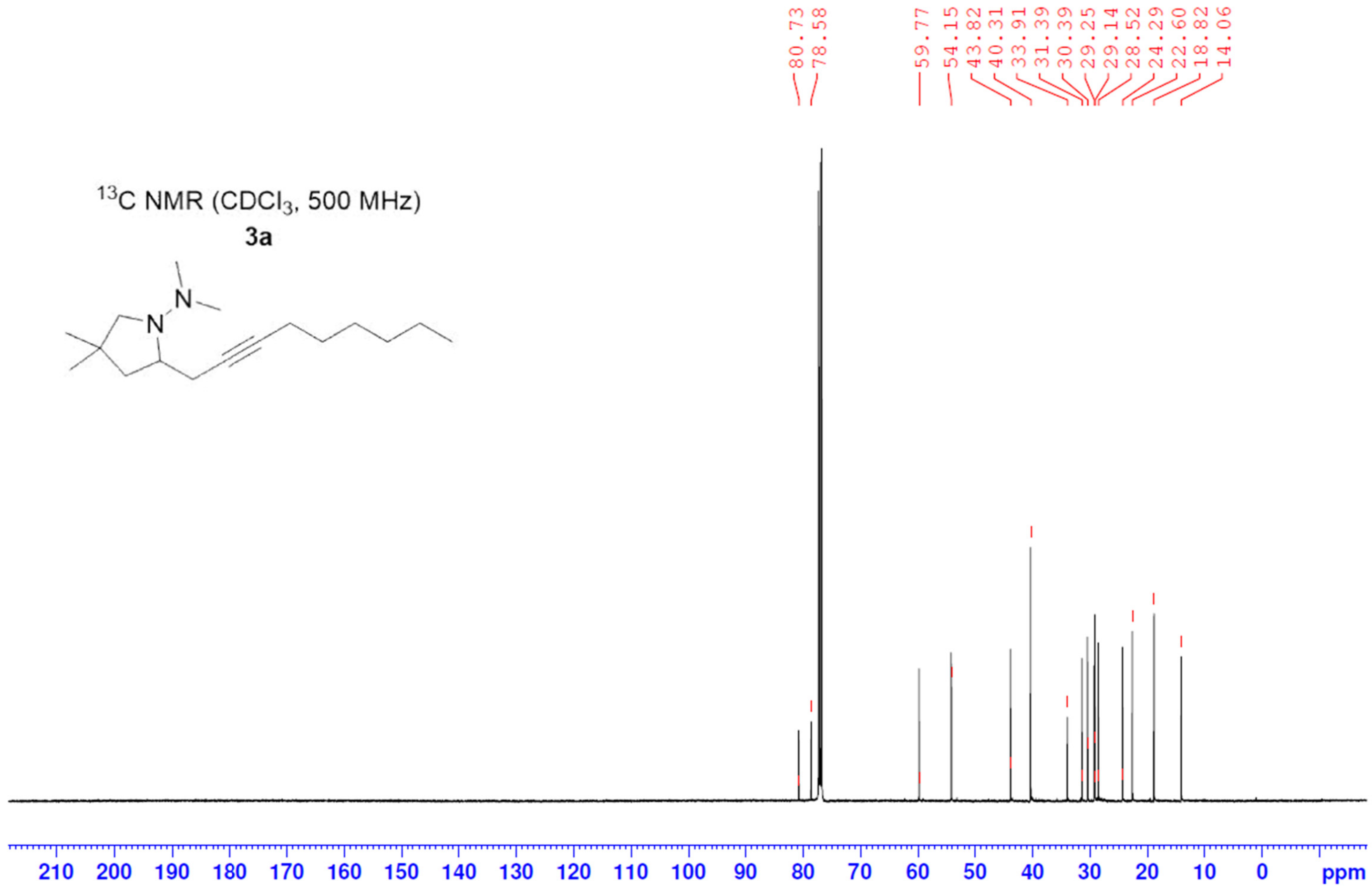

^1H NMR ($CDCl_3$, 500 MHz)

1b

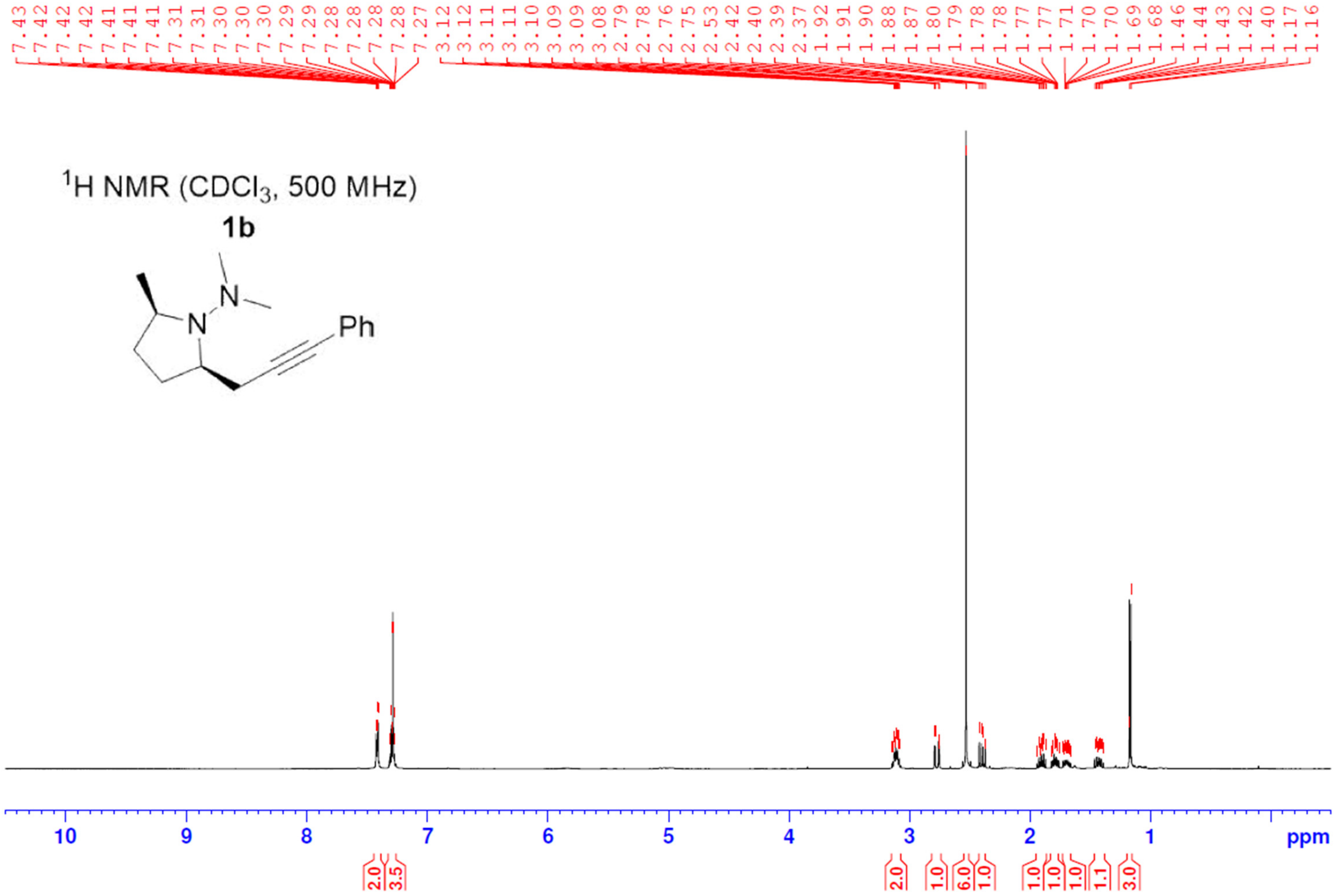

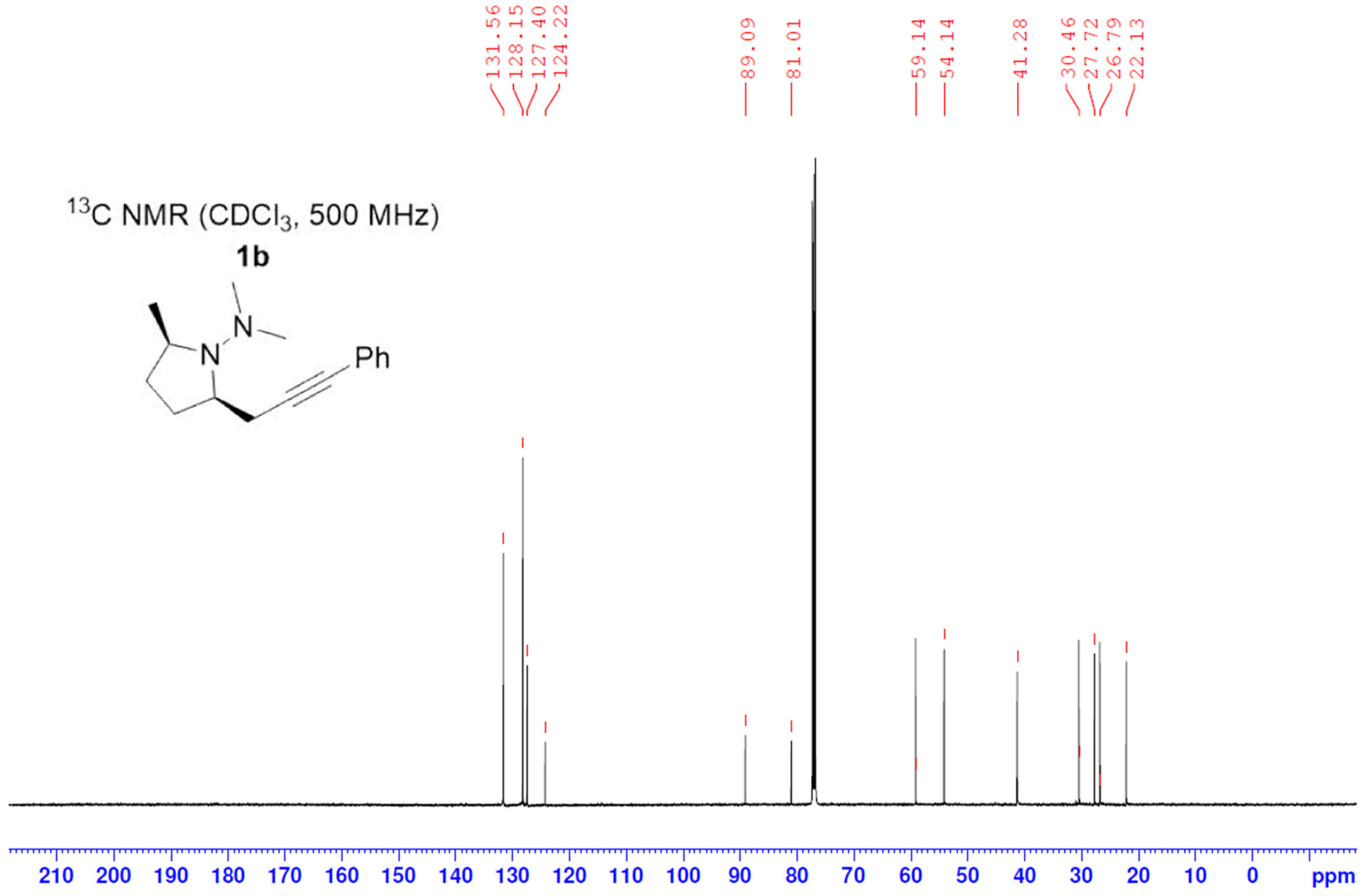

131.56
128.15
127.40
124.22
89.09
81.01
59.14
54.14
41.28
30.46
27.72
26.79
22.13
^{13}C NMR (CDCl$_3$, 500 MHz)
1b
N
N
Ph
210 200 190 180 170 160 150 140 130 120 110 100 90 80 70 60 50 40 30 20 10 0 ppm

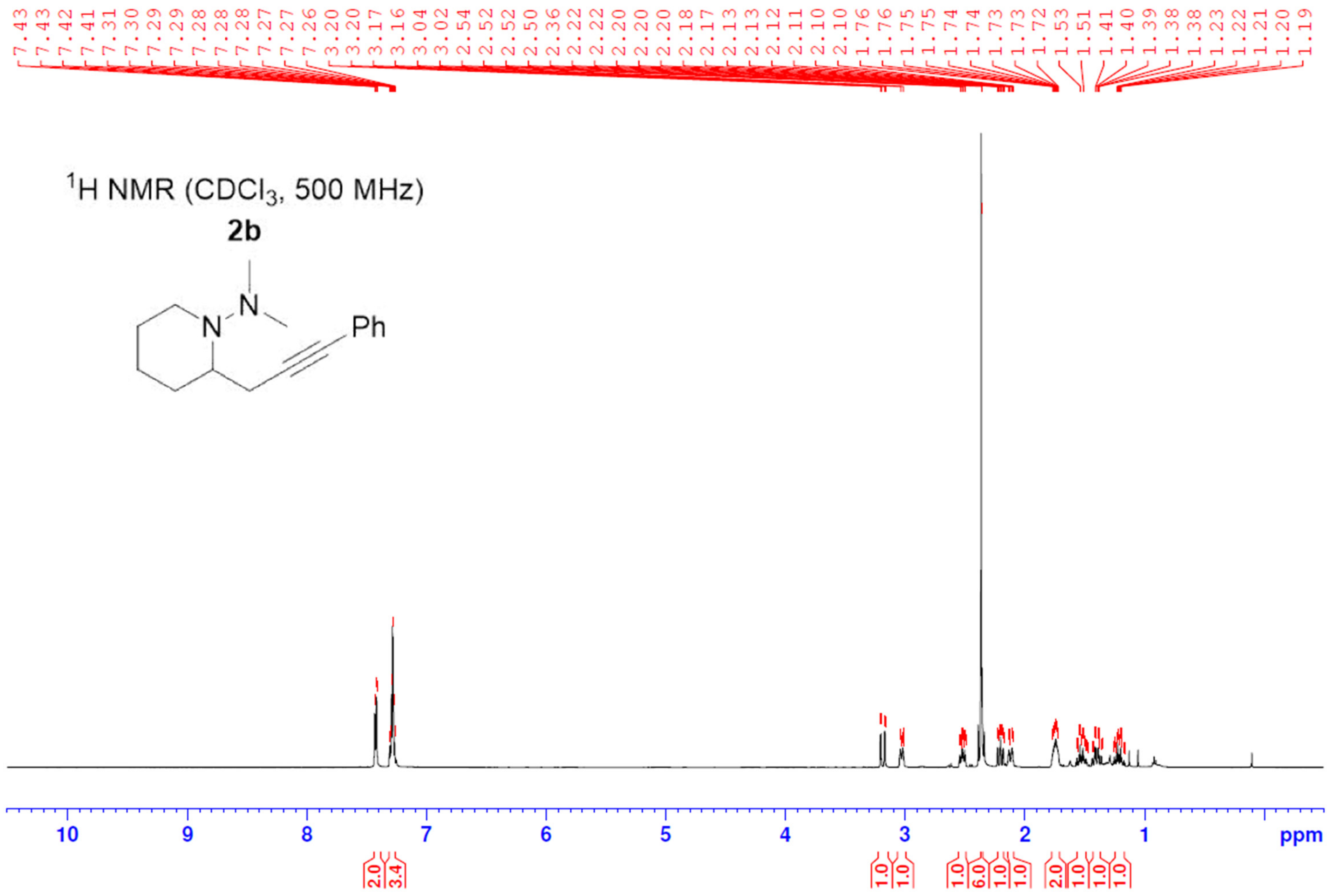
7.43 7.43 7.42 7.41 7.31 7.30 7.29 7.29 7.28 7.28 7.28 7.27 7.27 7.26 3.20 3.20 3.17 3.16 3.04 3.02 2.54 2.52 2.52 2.50 2.36 2.22 2.22 2.20 2.20 2.20 2.18 2.17 2.13 2.13 2.12 2.11 2.10 2.10 1.76 1.76 1.75 1.75 1.74 1.74 1.73 1.73 1.72 1.53 1.51 1.41 1.40 1.39 1.38 1.38 1.23 1.22 1.21 1.20 1.19
^{1}H NMR (CDCl$_3$, 500 MHz)
2b
N
N
Ph
10 9 8 7 6 5 4 3 2 1 ppm
2.0 3.4 1.0 1.0 1.0 6.0 1.0 1.0 2.0 1.0 1.0 1.0

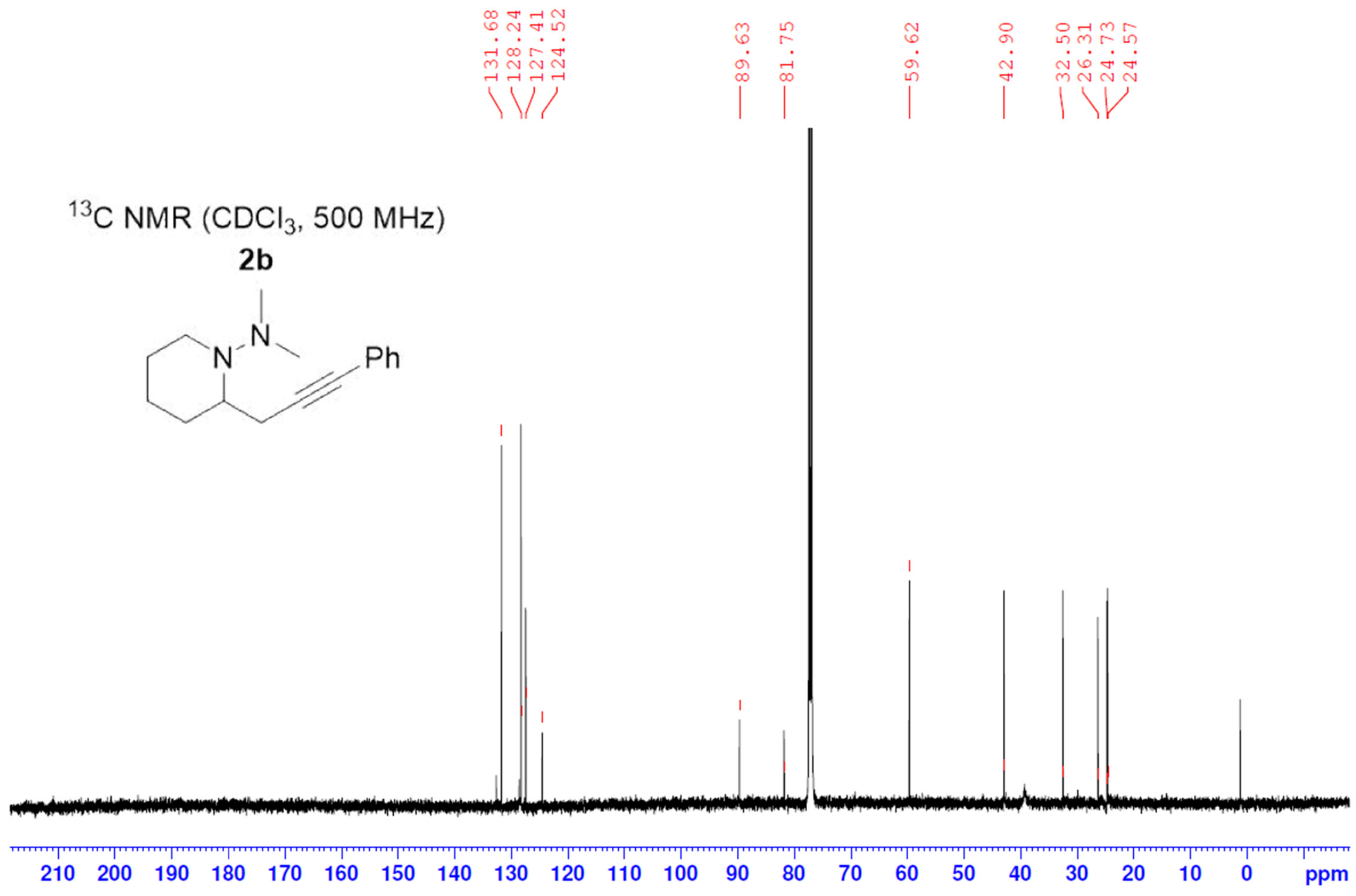

13C NMR (CDCl3, 500 MHz)
2b
N
N
Ph
131.68
128.24
127.41
124.52
89.63
81.75
59.62
42.90
32.50
26.31
24.73
24.57
210 200 190 180 170 160 150 140 130 120 110 100 90 80 70 60 50 40 30 20 10 0
ppm

S2:

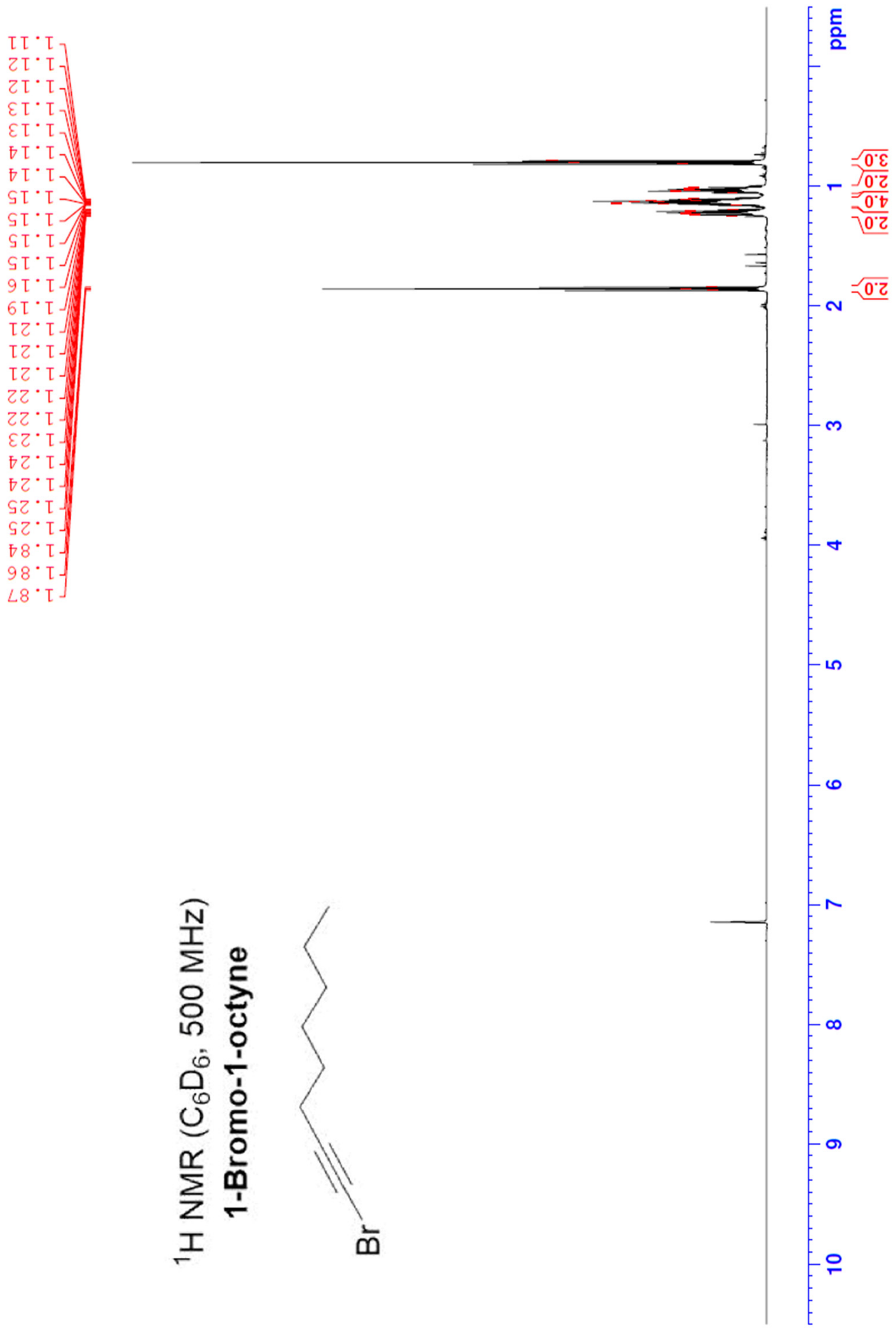

^1H NMR (C_6D_6, 500 MHz)
1-Bromo-1-octyne
Br
1.11
1.12
1.12
1.13
1.13
1.14
1.14
1.15
1.15
1.15
1.15
1.16
1.19
1.21
1.21
1.21
1.22
1.22
1.23
1.24
1.24
1.25
1.25
1.84
1.86
1.87
3.0
2.0
4.0
2.0
2.0
ppm

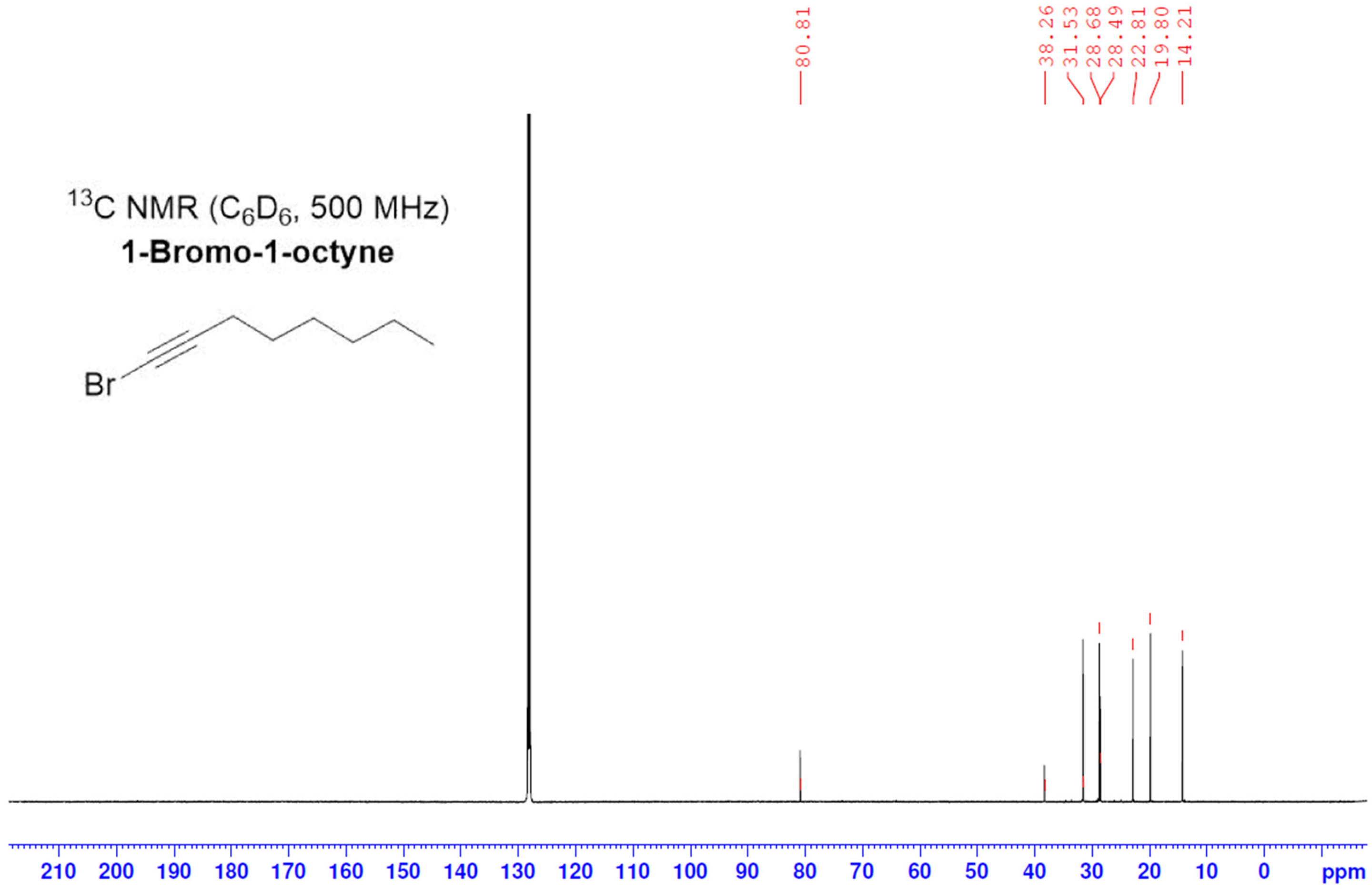

^{13}C NMR (C_6D_6, 500 MHz)
1-Bromo-1-octyne
Br
80.81
38.26
31.53
28.68
28.49
22.81
19.80
14.21
210 200 190 180 170 160 150 140 130 120 110 100 90 80 70 60 50 40 30 20 10 0 ppm

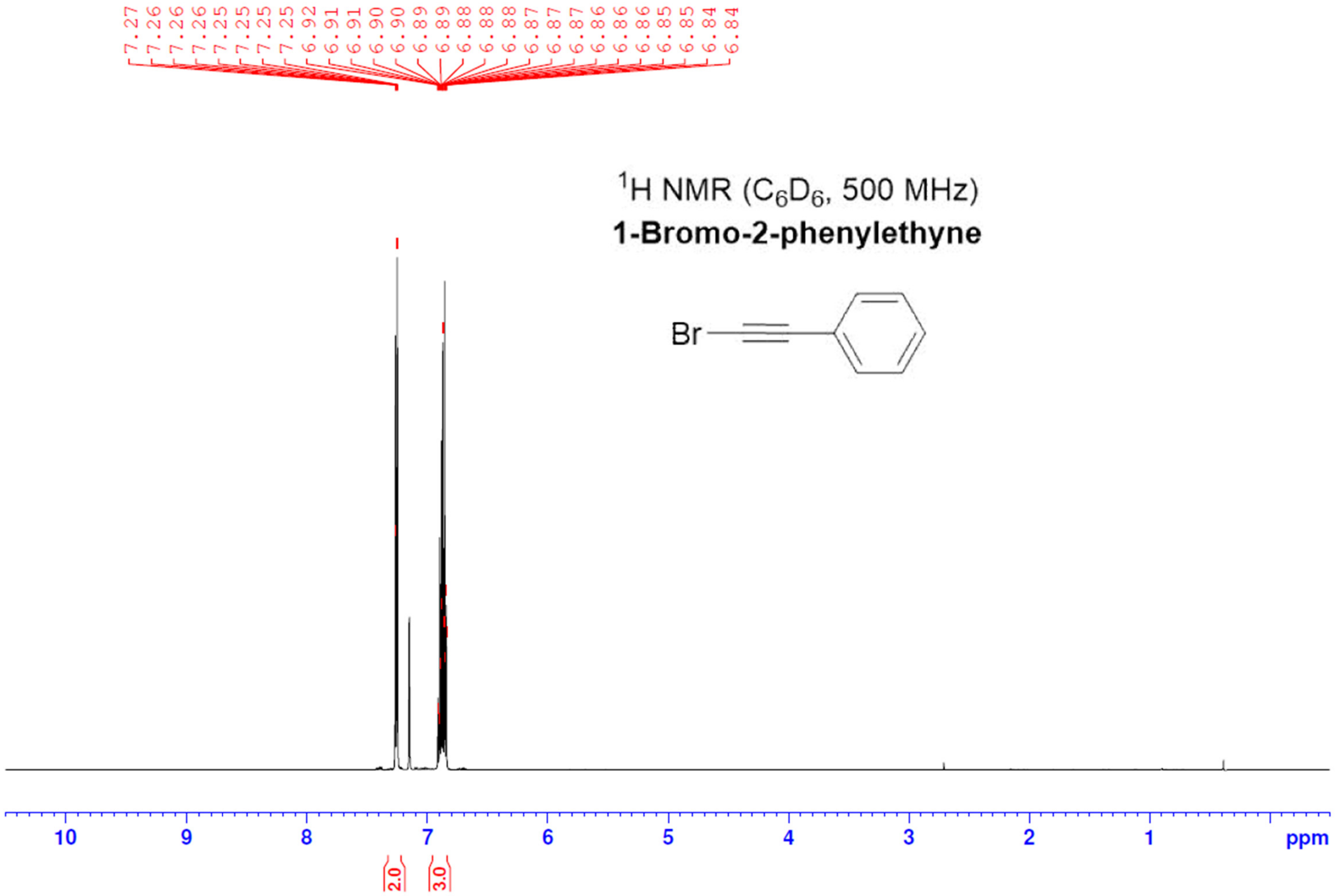
7.27
7.26
7.26
7.26
7.25
7.25
7.25
7.25
6.92
6.91
6.91
6.90
6.90
6.89
6.89
6.88
6.88
6.88
6.87
6.87
6.87
6.86
6.86
6.86
6.85
6.85
6.84
6.84
^{1}H NMR (C_6D_6, 500 MHz)
1-Bromo-2-phenylethyne
Br
10
9
8
7
6
5
4
3
2
1
ppm
2.0
3.0

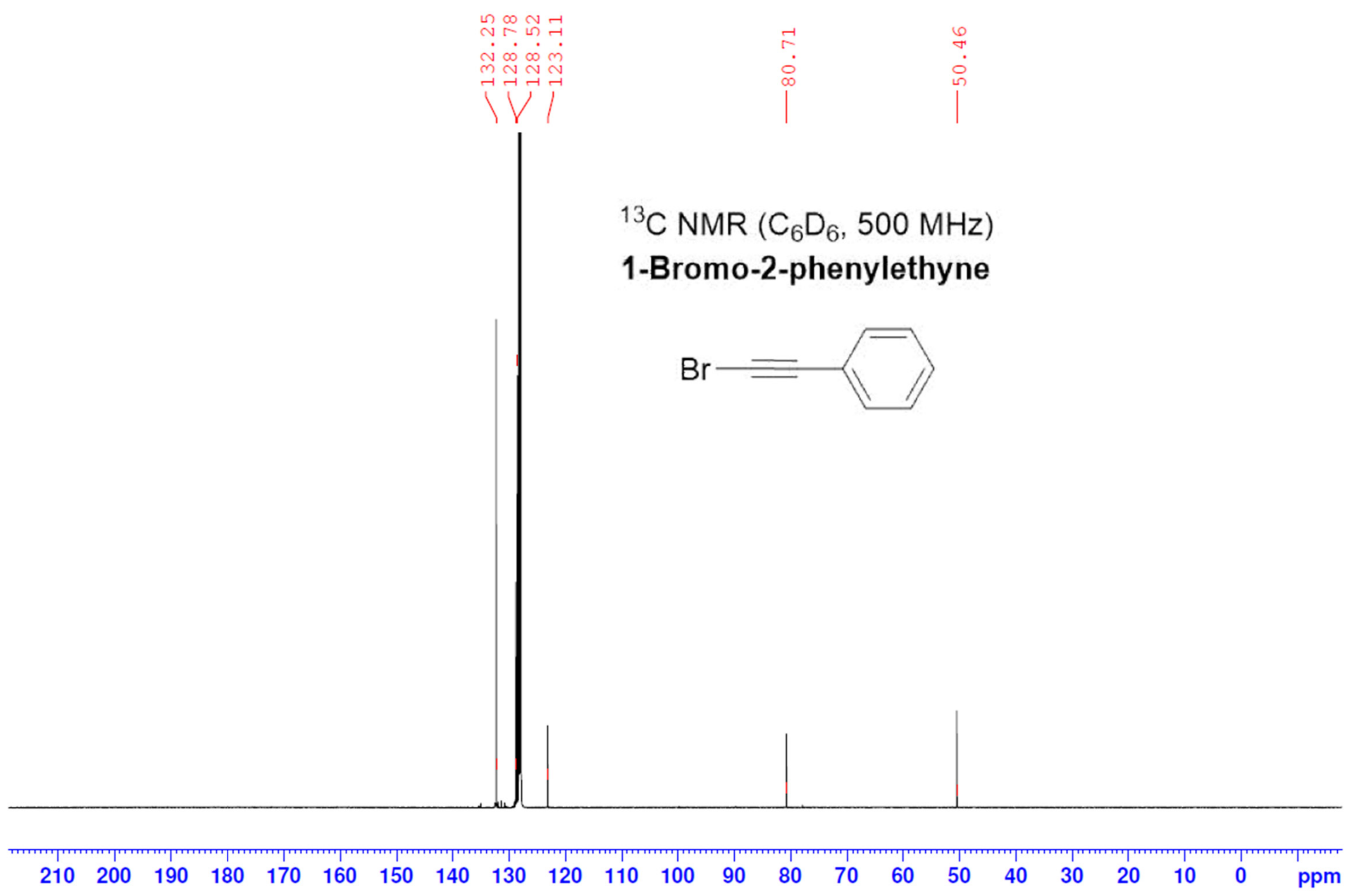

132.25
128.78
128.52
123.11
80.71
50.46
^{13}C NMR (C_6D_6, 500 MHz)
1-Bromo-2-phenylethyne
Br
210 200 190 180 170 160 150 140 130 120 110 100 90 80 70 60 50 40 30 20 10 0 ppm
